# Development and validation of a prediction model for suboptimal ovarian response in polycystic ovary syndrome (PCOS) patients undergoing GnRH-antagonist protocol in IVF/ICSI cycles

**DOI:** 10.1186/s13048-024-01437-w

**Published:** 2024-05-28

**Authors:** Xiaohang Xu, Yilin Jiang, Jinlin Du, Haoyue Sun, Xue Wang, Cuilian Zhang

**Affiliations:** 1 Reproductive Medical Center, People’s Hospital of Zhengzhou University, Zhengzhou, China; 2 Reproductive Medical Center, Henan Provincial People’s Hospital, Zhengzhou, China

**Keywords:** Predictive model1, Nomogram2, Polycystic ovary syndrome3, Ovarian response4, IVF/ICSI5

## Abstract

**Background:**

PCOS patients with unexpectedly low oocyte yield following conventional ovarian stimulation are referred to as suboptimal responders. However, identifying suboptimal responders presents a significant challenge within reproductive medicine and limited research exists on the occurrence of suboptimal response. This analysis aimed to develop a predictive model of suboptimal response during in vitro fertilization/intracytoplasmic sperm injection (IVF/ICSI) treatments in PCOS patients.

**Methods:**

This retrospective study involved a cohort of 313 PCOS patients undergoing their first IVF/ICSI cycle from 2019 to 2022. Univariate logistic regression analyses, least absolute shrinkage, selection operator regression analysis, and recursive feature elimination were employed to identify relevant characteristics and construct predictive models. Moreover, a nomogram was constructed based on the best model. Receiver operating characteristic curves, decision curve analysis (DCA), and calibration curves were used to evaluate the model.

**Results:**

The predictors included in the model were age, Anti-Mullerian hormone, antral follicle count, and basal follicle-stimulating hormone. The area under the receiver operating characteristic curve (AUC) was 0.7702 (95% confidence interval 0.7157–0.8191). The AUC, along with the DCA curve and calibration curve, demonstrated a satisfactory level of congruence and discrimination ability.

**Conclusion:**

The nomogram effectively predicted the probability of suboptimal response in PCOS patients undergoing gonadotropin-releasing hormone antagonist protocol during IVF/ICSI treatment.

## Introduction

Polycystic ovary syndrome (PCOS) is a prevalent and heterogeneous endocrine disorder that affects 6–15% of women of reproductive age [1, 2]. The condition is characterized by excess androgen levels, irregular menstrual cycles, polycystic ovarian morphology, insulin resistance (IR), elevated AMH levels, and elevated luteinizing hormone(LH)/ follicle-stimulating hormone(FSH) ratios, which affects affects follicle sensitivity to circulating gonadotropin(Gn), oocyte quality, ovulation, and lead to increased miscarriage rate and reproductive and metabolic dysfunction[ [2–6]].Controlled ovarian stimulation (COS) plays a pivotal role in obtaining a suitable number of oocytes and high-quality embryos to achieve satisfactory clinical outcomes in vitro fertilization-embryo transfer (IVF-ET) treatment. However, the ovarian response to exogenous gonadotropin (Gn) shows significant variation among PCOS patients [7, 8]. Some patients may exhibit a low number of retrieved oocytes, resulting in a reduced pregnancy rate and an elevated likelihood of cycle cancellation and miscarriage [9]. Therefore, identifying poor ovarian sensitivity in PCOS patients holds great significance in optimizing COS. In 2016, the “Patient-Oriented Strategies Encompassing Individualized Oocyte Number” (POSEIDON) criteria were proposed, and poor responders were categorized into four groups based on markers of ovarian reserve. Additionally, patients with good ovarian reserve were further classified into the poor response subgroup (< 4 oocytes) or suboptimal response subgroup (four to nine oocytes) based on the number of oocytes retrieved. Since the 1980s, gonadotropin-releasing hormone (GnRH) agonists have been utilized in COS to prevent premature LH surge through pituitary desensitization [10]. Alternatively, GnRH antagonist offers an instant blockade of LH secretion in the pituitary [11]. When comparing these two approaches, the GnRH antagonist protocol presents advantages, such as shorter treatment durations, reduced FSH stimulation time, and lower risk of ovarian hyperstimulation syndrome [12]. Consequently, the clinical application of the GnRH antagonist protocol has gained momentum. However, current methods to evaluate ovarian response of PCOS mainly rely on ovarian reserve markers, such as FSH, anti-Mullerian hormone(AMH), age, and antral follicle count(AFC). Yet, these indicators are insufficient for accurately identifying hyporesponders with normal ovarian reserve [13], especially in PCOS patients. Furthermore, it has been observed that 50 to 70% of PCOS patients exhibit insulin resistance [14]. The relationship between insulin and ovarian response is still controversial [15].Consequently, precise methods to predict suboptimal response to the GnRH antagonist protocol in PCOS patients remain lacking. Therefore, the objective of this study was to screen out the factors that can predict suboptimal ovarian response in the GnRH antagonist protocol and develop an individualized prediction model, in order to implement personalized ovulation strategies for PCOS patients.

## Materials and methods

### Subjects

**Fig. 1:**
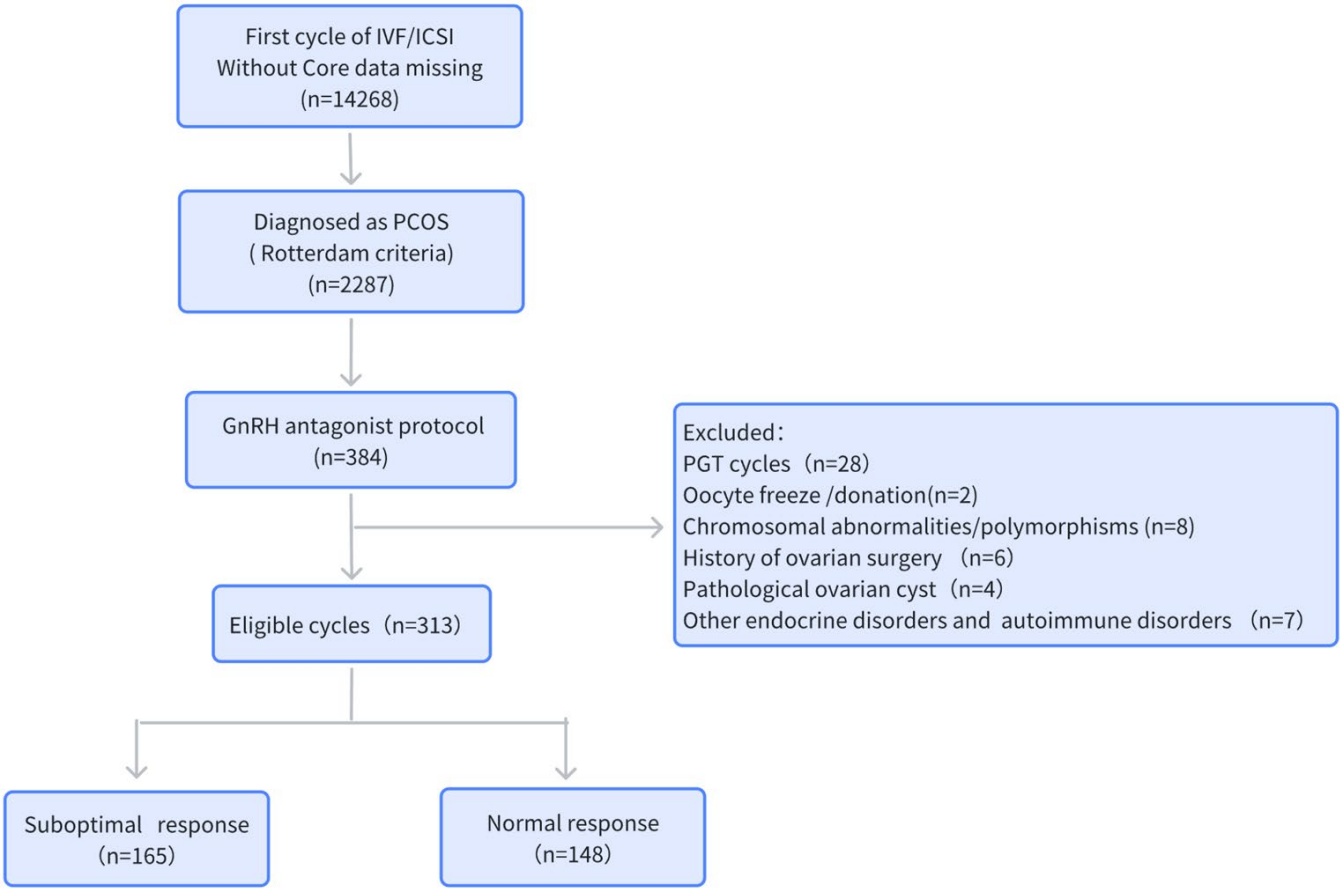
Flowchart of the data collection process *Abbreviations* IVF/ICSI: in vitro fertilization/intracytoplasmic sperm injection; PCOS, polycystic ovary syndrome; GnRH, gonadotropin-releasing hormone; PGT, preimplantation genetic testing

A retrospective observational cohort analysis was conducted in patients who underwent their first cycle of IVF or intracytoplasmic sperm injection (ICSI) treatment in Henan Provincial People’s Hospital between January 2019 and December 2022. The criteria for inclusion were as follows: (1) First cycle of IVF/ICSI treatment with complete key records; (2) Diagnosed with PCOS according to the Rotterdam criteria [16];(3) GnRH antagonist protocol; The exclusion criteria were: (1) Preimplantation genetic testing cycle; (2) Cycles involving oocyte cryopreservation or donation; (3) Female chromosomal abnormalities or chromosomal polymorphisms; (4) Coexisting factors that may affect ovarian response, such as history of ovarian surgery or pathological ovarian cyst; (5)Metabolic disorders: diabetes, hypertension, hyperprolactinemia, hyperthyroidism, hypothyroidism and autoimmune disorders. The patient inclusion and exclusion process is presented in Fig. 1.

In this study, patients with no more than 9 retrieved oocytes were categorized into the suboptimal ovarian response (SOR group), while the remaining patients were categorized into normal ovarian response group (NOR group).

### Data acquisition

The primary data for this study were collected from the electronic medical record system of our hospital, encompassing all assisted reproductive data. This analysis was authorized by the Henan Provincial People’s Hospital Ethics Committee (No. 2,022,139).

The basal sex hormone was measured on days 2–4 of the menstrual cycle. Anti-Mullerian hormone (AMH), fasting blood glucose, and insulin levels were measured prior to COS at our center using an ADVIA2400 Chemistry System (ADVIA 2400, SIEMENS, Germany) and a chemiluminescence immunoassay analyzer (Cobas8000 e602; Roche Diagnostics GmbH, Mannheim, Germany). The inter-assay coefficient of variation (CV) in the laboratory was lower than 3.5%. Our laboratory undergoes annual qualification checks by the External Quality Assessment of Clinical Laboratory Center under the Ministry of Health of the People’s Republic of China in Beijing. Patients with fasting blood glucose ≥ 6.1mmol/L or hyperinsulinemia would undergo an OGTT test and insulin release test, and receive counseling from the endocrinology department. The AFC was assessed by transvaginal ultrasonography (Voluson E8 Expert; GE Healthcare, Chicago, ) with a 4- to 9-MHz probe (RIC5-9-D Endocavity transducer; GE Healthcare, Chicago) by a relatively fixed group of experienced reproductive physicians. AFC was defined as the total count of follicles measuring 2–9 mm in diameter in both ovaries. The homeostasis model assessment of insulin resistance (HOMA-IR) = fasting blood glucose (mmol/L)× fasting insulin (µU/mL)/22.5 [17]. Follicle to Oocyte Index (FOI) = oocytes retrieved /(AFC). Pregnancy outcome data were collected until October 2023. The live birth rate was defined as the live birth per embryo transfer cycle. The cumulative pregnancy rate was defined as the total number of pregnancies achieved across all ET cycles in one retrieval cycle.

### Ovarian stimulation protocol, embryo transfer and luteal support

The COS protocols and Gn dosage were customized based on the patient’s age, weight, and ovarian reserve using a step-up regimen for the Gn dose. In the GnRH antagonist flexible protocol, Gn was injected from day 2–3 of the menstrual cycle and GnRH antagonist (Cetrotide; 0.25 mg) was added daily from day 6–7 of stimulation upon detection of one dominant follicle ≥ 12 mm. When one dominant follicle ≥ 20 mm or three follicles ≥ 17 mm or 2/3 follicles ≥ 16 mm were found, recombinant/ urinary human chorionic gonadotropin (hCG) was injected. Transvaginal ultrasound-guided aspiration was conducted 35 ∼ 38 h following hCG injection to pick up oocytes. The Istanbul consensus scoring system was applied to evaluate embryos [18]. A transferable embryo on day 3 referred to an embryo with ≥ 4 cells, < 26% fragmentation, and either no asymmetry or moderate asymmetry, which could be transferred or cryopreserved. Embryo transfer was performed 3 or 5 days after oocyte retrieval. The endometrial preparation protocol was selected individually in frozen embryo transfer cycles. One or two cleavage embryos or blastocysts were transferred. Vaginal combined oral progesterone was administered for luteal-phase support until 8 ∼ 10 weeks.

### Statistical analyses

Data analysis was performed using EmpowerStats statistical software (X&Y Solutions) based on R software (http://www.R-project.org, the R Foundation). Continuous variables were reported as mean ± standard deviation (SD), while categorical variables were expressed as N (%). Univariable logistic regression analyses were carried out to identify the relevant factors for suboptimal ovarian response. The least absolute shrinkage and selection operator (LASSO) binary logistic regression analyses and recursive feature elimination (RFE) were implemented to select variables and rank the variable importance. Additionally, 5-fold cross-validation was utilized as a resampling method. The Akaike information criterion (AIC) [19] and receiver operating characteristic (ROC) curve were employed to compare the predictive capability among the models. Furthermore, a nomogram was created using the “rms” package for the best-performing model to provide graphical representations and facilitate users in calculating probabilities [20]. ROC curve, decision curve analysis (DCA) curve and calibration curve were used to evaluate the nomogram.

## Results

### Baseline characteristics

This study included a total of 313 patients, comprising 165 cases (52.72%) in the suboptimal ovarian response (SOR) group and 148 (47.28%) cases in the normal ovarian response (NOR) group. A total of 17 cycles were canceled due to a lack of transferable embryos and 133 cycles underwent fresh embryo transfer. Table 1 outlines the baseline characteristics. Significant differences were observed in terms of age, basal level of follicle-stimulating hormone (bFSH), luteinizing hormone (LH)/FSH ratio, testosterone (T), AMH, fasting insulin, the initial dosage of Gn, and Gn duration between the two groups (*P* < 0.05). No statistical significance was observed in weight, BMI, infertility type, infertility duration, basal level of LH, estrogen (E2), progesterone (P), prolactin (PRL) progesterone (P), AFC, fasting blood glucose, HOMA-IR, and total Gn usage (*P* > 0.05).

**Table 1:** Comparison of the basic parameters of the study population by univariable logistic regression analysis

	SOR group	NOR group	*P*-value
N	165	148	
Age	31.32 ± 4.66	30.01 ± 4.05	**0.009**
Weight	61.01 ± 9.90	60.67 ± 10.79	0.774
BMI	23.40 ± 3.42	23.41 ± 4.13	0.990
Primary fertility			0.699
Yes	85 (51.52%)	73 (49.32%)	
No	80 (48.48%)	75 (50.68%)	
Infertility years	3.58 ± 2.67	3.31 ± 2.35	0.351
Basal FSH	6.86 ± 1.72	5.73 ± 1.35	**< 0.001**
Basal LH	5.64 ± 3.53	5.85 ± 3.36	0.596
BasaLH/FSH	0.85 ± 0.54	1.04 ± 0.59	**0.004**
Basal prolactin	16.64 ± 7.74	16.84 ± 7.26	0.832
Basal estrogen	40.65 ± 17.32	39.33 ± 15.65	0.492
Basal testosterone	0.29 ± 0.19	0.34 ± 0.17	**0.041**
Basal progesterone	0.31 ± 0.20	0.32 ± 0.18	0.564
AMH	3.61 ± 2.38	5.39 ± 3.16	**< 0.001**
AFC	23.68 ± 3.59	23.61 ± 4.00	0.869
Glucose	4.88 ± 0.49	4.85 ± 0.53	0.589
Insulin	13.43 ± 7.26	11.94 ± 5.55	**0.045**
HOMA-IR	2.95 ± 1.76	2.61 ± 1.34	0.056
Initial Dosage of Gn	197.20 ± 58.93	168.50 ± 51.45	**< 0.001**
Total Gn dosage	1896.98 ± 636.64	1759.63 ± 758.59	0.083
Gn duration	8.71 ± 2.42	9.26 ± 2.39	**0.046**

Values are presented as mean ± standard deviation or number (percentage)

*Abbreviations* FSH, follicle-stimulating hormone ; LH, luteinizing hormone; AMH, anti-Mullerian hormone; AFC, total antral follicle count; BMI, body mass index; HOMA-IR, homeostasis model assessment of insulin resistance; Gn: gonadotropin;

### Analysis of patients’ clinical outcomes

The SOR group exhibited a significantly lower level of dominant follicle count, estrogen on the hCG trigger day, retrieved oocytes, FOI, MII oocytes, 2PN embryos, and transferable embryos compared to the POR group (*P* < 0.05). In contrast, no statistically significant difference was observed in HCG day endometrial thickness and clinical outcomes, such as pregnancy rate, ectopic rate, miscarriage rate, and live birth rate of the fresh cycle between the two groups (*P* > 0.05). The CCPR was lower in the SOR group but showed no statistically significant difference from the NOR group, as shown in Appendix Table A1

### Parameter selection and model building

**Fig. 2:**
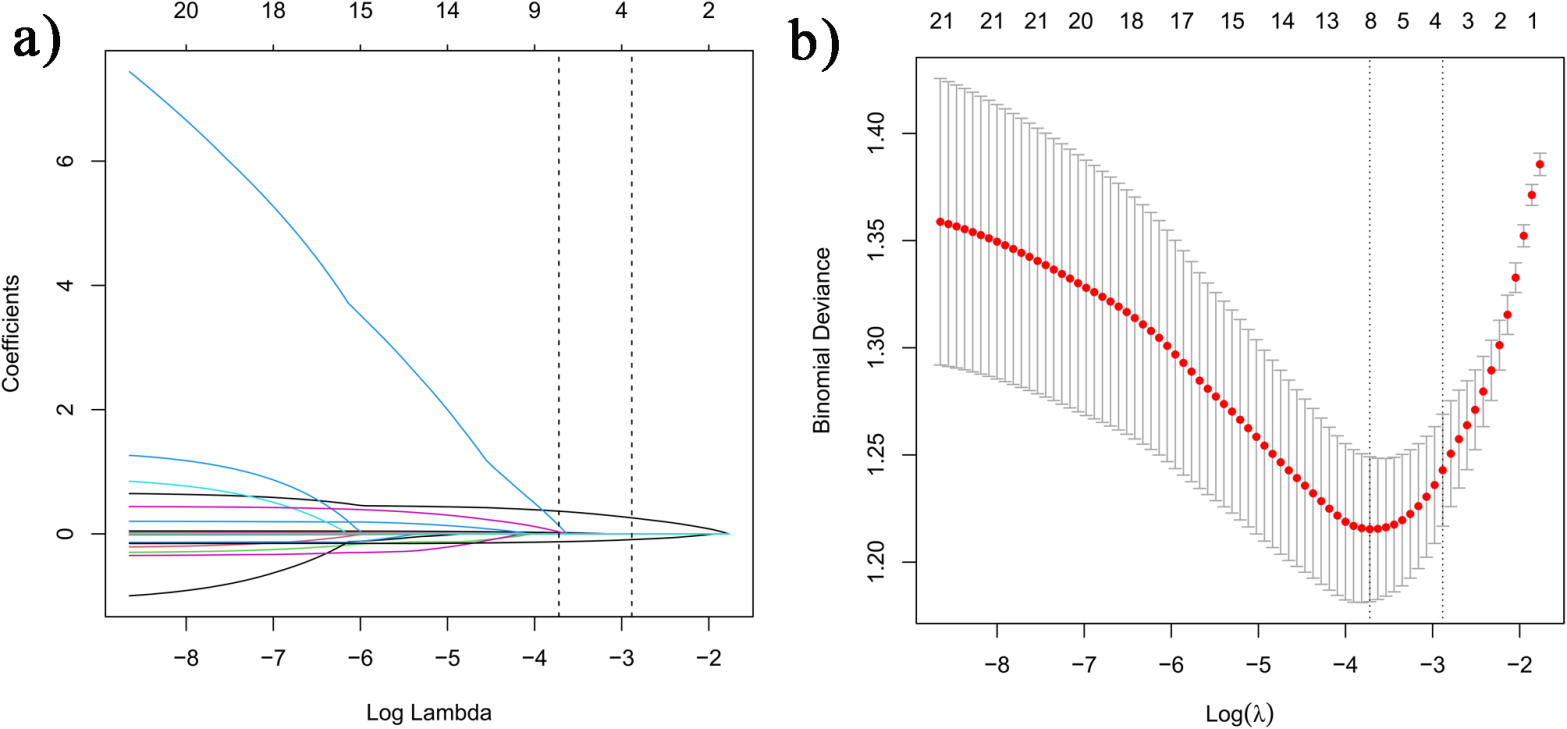
Variable selection using the least absolute shrinkage and selection operator (LASSO) regression algorithm.(**a**)Lasso regression path diagram; (**b**) LASSO coefficient profiles of the characters. Parameters were screened out by 10-fold cross-validation and using lambda-1se as criteria

LASSO regression and RFE algorithm were adopted to screen out the associated factors for suboptimal response, which were considered ideal methods for conducting interaction testing, variable selection, and parameter estimation without overfitting. A total of 20 potentially related variables (listed in Table 1) were analyzed and four variables were ultimately identified as predictors (age, AMH, bFSH, insulin) of suboptimal response by LASSO regression, as shown in Fig. 2a and b. In addition, 16 variables were selected after near zero variance and collinearity check, and the importance ranking was determined by RFE. Subsequently, different strategies were adopted to construct models. Model 1 (AMH, basal FSH, insulin, initial dosage of Gn) included the variables identified by LASSO regression. Model 2 (age, AMH, basal FSH, insulin) incorporated the top 4 features that were obtained before COS. Model 3 further integrated COS parameters (Gn duration, initial dosage of Gn, total Gn dosage) in addition to those in Model 2. Finally, Model 4 encompassed all the 16 selected indicators, as presented in Table 2.

**Table 2:** Variable Importance ranking and model building

	Variable importance	Model1 ( LASSO regression selected)	Model2 ( pre-COS variables)	Model3 ( pre-COS + COS variables)	Model4 (All variables)
Basal FSH	100.00	Y	Y	Y	Y
Insulin	72.78	Y	Y	Y	Y
AMH	55.80	Y	Y	Y	Y
Age	28.00		Y	Y	Y
Gn duration	17.50			Y	Y
>Total Gn dosage	12.24			Y	Y
AFC	11.77				Y
Basal E2	11.06				Y
Infertility years	11.06				Y
Basal P	9.72				Y
Weight	4.43				Y
Initial dosage of Gn	2.52	Y		Y	Y
Basal PRL	1.85				Y
Basal LH	1.28				Y
Glucose	0.38				Y
BMI	0.00				Y

### Evaluation of different prediction models

**Fig. 3:**
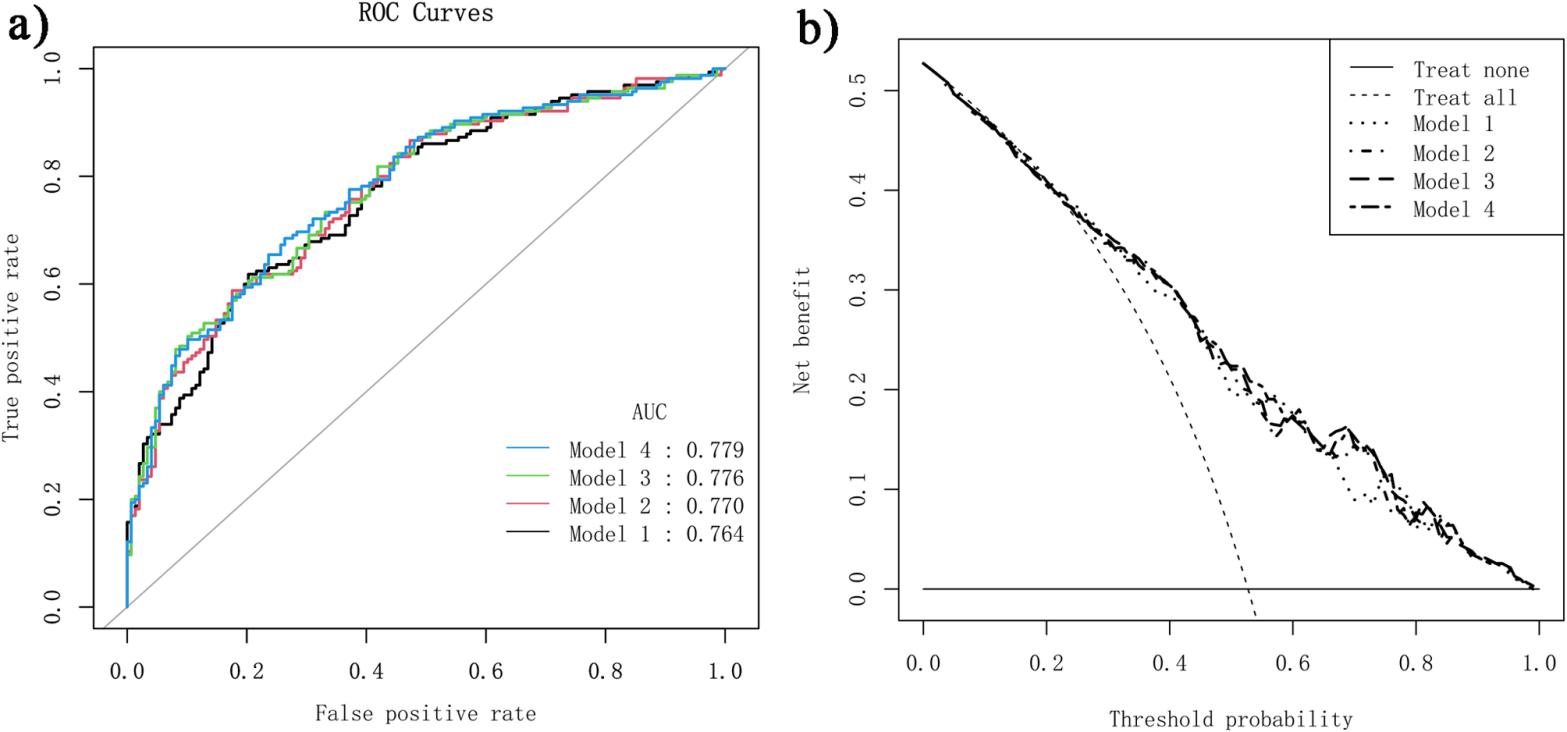
(**a**) Receiver operating characteristic (ROC) curve of the four predictive models; (**b**) Decision curve analysis (DCA) of the predictive models for suboptimal response

The discriminatory abilities, ROC curves, and DCA curves of the above models are listed in Table 3 and Fig. 3. When comparing the parameters related to predictive ability listed in Table 3, these four models exhibit similar predictive performance with regard to accuracy, sensitivity, specificity, recall, and F1 score. Model 2, which solely incorporates pre-COS indicators, exhibited the lowest AIC, along with comparable ROC curves and DCA curves among the four models. After a comprehensive comparison of predicton performance and clinical convenience, Model 2 was selected for assessing suboptimal ovarian response. This model includes age, AMH, bFSH, and insulin as indicators. The equation for the predictive model was as follows: logit (suboptimal response) = -4.89032 + 0.05989*age − 0.18735*AMH + 0.49104*bFSH + 0.07123*Insulin.

**Table 3:** Performance of four predictive models

	Model 1	Model 2	Model 3	Model 4
Accuracy (95%CI)	0.668 (0.613, 0.720)	0.693 (0.639, 0.744)	0.696 (0.642, 0.747)	0.693 (0.639, 0.744)
Accuracy Null	0.527	0.527	0.527	0.527
P-value (Accuracy > Null)	0.000	0.000	0.000	0.000
Sensitivity	0.6970	0.7576	0.7394	0.7394
Specificity	0.6351	0.6216	0.6486	0.6419
Positive Prediction Value	0.6805	0.6906	0.7011	0.6971
Negative Prediction Value	0.6528	0.6970	0.6906	0.6884
Precision	0.6805	0.6906	0.7011	0.6971
Recall	0.6970	0.7576	0.7394	0.7394
F1	0.6886	0.7225	0.7198	0.7176
Area under curve (AUC)	0.7642	0.7702	0.7758	0.7789
AIC	372.02	370.94	374.23	384.82

Model 1 (LASSO regression selected) : AMH, Basal FSH, insulin, initial dosage of Gn

Model 2 ( pre-COS variables): age, AMH, basal FSH, insulin

Model 3 ( pre-COS + COS variables): Age, AMH, basal FSH, insulin, Gn duration, initial dosage of Gn, Total Gn

Model 4 (All variables) : AFC, Age, AMH, bFSH, Basal P, Basal PRL, Basal T, BMI, Gn duration, initial dosage of Gn, Infertility years, Insulin, Total Gn

### Development and validation of the nomogram

**Fig. 4:**
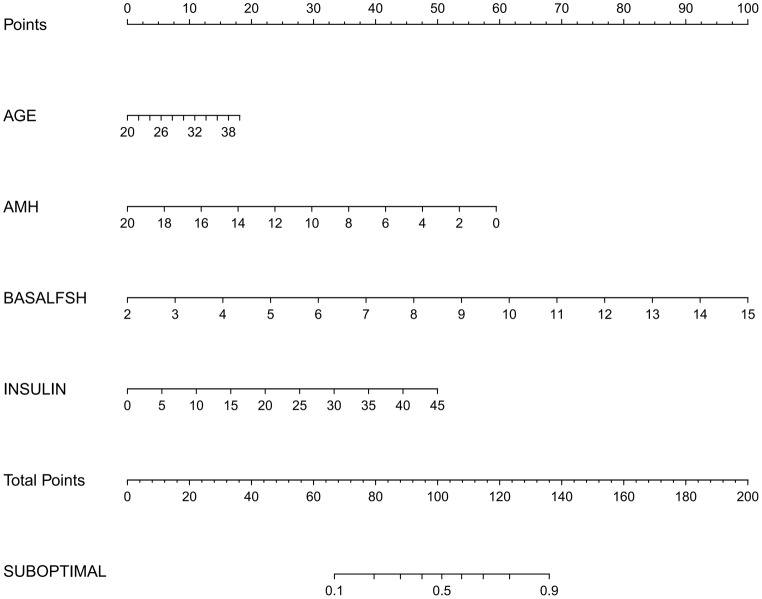
Nomogram for predicting the suboptimal response of PCOS patients. The points for each variable were calculated by drawing a vertical line from the value to the axis labeled “Total Points”. The total score corresponds to the probability of a suboptimal response in the lowest axis

The nomogram of the prediction model is depicted in Fig. 4. Each parameter was assigned a vertical extension (refer to the top points bar) individually. The total score was acquired by summing up the scales of each factor. The overall point projected on the bottom scale suggests the likelihood of a suboptimal response.

**Fig. 5:**
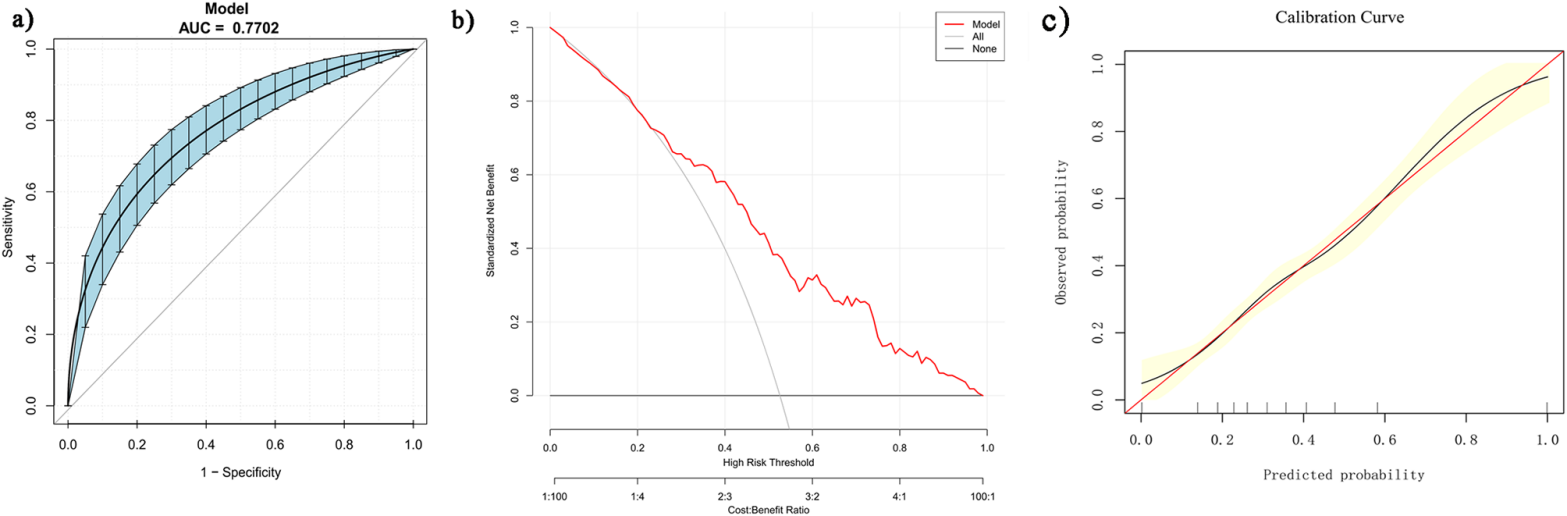
Validation of the nomogram for suboptimal response. (**a**) Receiver operating characteristic (ROC) curve of the nomogram (resampling times = 500); (**b**) Decision curve analysis (DCA) of the predictive model; (**c**) Calibration curve of the nomogram

The discriminatory abilities, goodness of fit, and clinical validity of the nomogram are presented in Fig. 5. The bootstrap method was performed for inner validation and the model demonstrated good discriminative potential, with an AUC of 0.7702 (95% confidence interval(CI): 0.7157–0.8191, resampling times = 500). The calibration curve for this nomogram showed a good consistency between predicted and actual probability and the DCA curve demonstrated a satisfactory level of clinical usefulness of the nomogram.

## Discussion

A precise forecast of ovarian response is essential for successful ovarian stimulation in PCOS patients. Ovarian reactivity determines the ability to recruit appropriate number of oocytes, which is a key factor for the success of COS [21]and directly affects the outcome of assisted reproduction. The GnRH antagonist protocol can rapidly reduce endogenous LH and FSH levels by competitively binding to endogenous GnRH receptors, effectively inhibiting the LH peak. This procedure has become a mainstream clinical program, especially in PCOS [22],due to its advantages such as convenient use, flexibility, lower Gn consumption, shorter stimulation duration, fewer occurrence of ovarian hyperstimulation syndrome and higher patient satisfaction [12]. However, the complexity of ovarian response among PCOS women presents a challenge in prescribing a suitable initial dose of Gn [23]. Therefore, identifying patients who are at risk of hyporesponse holds clinical significance. Currently, most prediction models focus on hyperesponse [23], and no consensus has been reached to identify PCOS patients with a suboptimal response before COS; furthermore, no acknowledged mathematical model has been developed to predict such patients. Hence, our study developed and validated a predictive model for suboptimal response. To our knowledge, this is the first attempt to predict suboptimal response in PCOS patients undergoing the GnRh antagonist protocol. This study adopted diverse parameter selection methods and established variable importance rankings. Subsequently, four models were constructed to validate the predictive performance. A balance between predictive efficacy and clinical convenience was achieved. The model included the top 4 baseline indicators of PCOS patients, namely age, AMH, basal FSH, and insulin, for estimating the suboptimal ovarian response in PCOS. The equation for the predictive model was logit(suboptimal response) = -4.89032 + 0.05989*age − 0.18735*AMH + 0.49104*bFSH + 0.07123*Insulin. Meanwhile, the prediction model was illustrated as a nomogram, which can be applied in patient counseling and clinical decision-making before COS.

Consistent with multiple studies, our research revealed that ovarian reserve markers (bFSH, AMH, age) can serve as predictive factors to identify PCOS patients at risk of suboptimal response. The association between ovarian reserve markers such as age, AMH, and bFSH and the ovarian response has been reported in patients with normal ovarian reserve [23].In addition, many studies have uncovered the remarkable precision of ovarian reserve markers in predicting poor ovarian response [24] and have established mathematical models to forecast ovarian sensitivity [25, 26]. In infertile PCOS women receiving letrozole for ovulation induction, increased levels of baseline LH/FSH ratio, AMH, and free androgen index were found to be negatively associated with response to letrozole treatment [27]. Moreover, PCOS individuals with significantly higher circulating AMH levels were more likely to be resistant to exogenous Gn ovulation induction and required a higher starting dosage [28]. Chen et al. discovered that serum AMH levels were an independent indicator of oocyte number in PCOS among different protocols [29]. Notably, although ovarian reserve markers possess high sensitivity, their reliability is not absolute, with false positive rates ranging from 10–20% [23]. To mitigate this issue, our model incorporates multiple parameters to reduce the potential for misinterpretation; therefore, the most important ovarian reserve markers were included.

Our study also indicated that insulin is associated with suboptimal response. Hence, insulin was incorporated into the predictive model. This is the primary innovation of our research and the main distinction from the previous model for assessing ovarian sensitivity. Insulin resistance may directly impact oocyte maturation and ovulation in PCOS patients [30, 31]. Li et al. discovered a negative correlation between HOMA-IR and ovarian response in PCOS patients [32]. Luo et al. also observed that insulin resistance decreased the ovarian sensitivity index in PCOS patients, particularly in the non-overweight subgroup [15]. Emerging evidence has described the interplay between insulin resistance and atresia of antral follicles in PCOS [33, 34]. Nonetheless, the mechanisms underlying the declining ovarian sensitivity during COS have not been fully explained. Insulin may play a pivotal role in ovarian function and may be implicated in promoting follicle development [35]. Elevated insulin levels, especially in obese sufferer, stimulate the generation of androgens in ovary by facilitating P450 c17α enzyme synthesis, as well as LH receptor expression on theca cells [36, 37], resulting in increased estrogen and exerting a negative impact on the hypothalamic-pituitary-ovarian axis. Consequently, this can inhibit follicle growth by suppressing FSH secretion and prolonging the duration of ovarian stimulation [38]. Fortunately, insulin resistance is reversible. Given the higher risk of poor response, insulin management should be encouraged to potentially improve clinical outcomes.

Nevertheless, the limitations of the present study should be acknowledged. First, the inherent biases associated with retrospective analysis may impact the results. To mitigate selection bias, this study focuses on PCOS patients undergoing the Gnrh-antagonist protocol and sets relatively broad criteria for age, ovarian reserve, and BMI. Individuals with untreated metabolic or endocrine abnormalities, pathological ovarian cysts, or a history of ovarian surgery were excluded, which may potentially influence the ovarian response. These inclusion and exclusion criteria were employed to ensure population homogeneity and enhance practical clinical applicability. Furthermore, limited by the data collection, the metformin dosage in patients with insulin resistance and post-treatment insulin levels were not available. However, our center adopted standardized treatment for patients with insulin resistance without any bias across different populations. The majority of patients had their insulin levels measured within one month prior to COS, and we believe that the pre-ovulation insulin level can serve as a reliable indicator of the patient’s insulin resistance status. Therefore, additional research with a larger, diverse sample from multiple reproductive centers is necessary for external validation. These findings will assist in creating and validating a more robust prediction model. Additionally, machine learning can be utilized to enhance the prediction model using extensive data, allowing for continual improvement and broader application to relevant populations. This predictive model has the potential to identify PCOS patients at high risk of suboptimal response at an early stage, enabling lifestyle counseling and timely medication adjustments to reduce low response rates and enhance clinical outcomes.

## Conclusions

In summary, our study has developed a predictive model incorporating age, AMH, bFSH, and insulin as indicators to estimate the probability of suboptimal response in PCOS patients undergoing the GnRH-antagonist protocol. Our model can assist in making clinical decisions.

## Data Availability

The data presented in this study are available on request from the corresponding author.The data are not publicly available due to participant privacy.

## Appendix A

**Table A1:** Comparison of clinical outcomes by univariable logistic regression analysis

	SOR group	NOR group	*P*-value
N	165	148	
HCGdayfollicle	6.08 ± 3.31	11.97 ± 5.16	< 0.001
HCG day Estrogen	1288.86 ± 748.68	2152.83 ± 1151.86	< 0.001
HCG day Endometrial thickness	10.46 ± 2.53	10.67 ± 2.47	0.457
Oocyte	6.07 ± 2.10	16.32 ± 6.40	< 0.001
FOI(Follicle to Oocyte Index)	0.53 ± 0.33	1.00 ± 0.46	< 0.001
No of MII	5.04 ± 2.23	13.14 ± 6.04	< 0.001
No of 2PN	3.40 ± 2.08	9.47 ± 4.92	< 0.001
Transferable Embryos on D3	2.32 ± 1.49	5.58 ± 3.45	< 0.001
Clinical pregnancy rate of fresh cycle			0.859
Yes	43 (61.43%)	37 (58.73%)	
No	27 (38.57%)	26 (41.27%)	
Miscarriage rate of fresh cycle			0.532
Yes	5(11.63%)	4(10.81%)	
No	38(88.37%)	33(89.19%)	
Ectopic pregnancy rate of fresh cycle			0.999*
Yes	3 (7.00%)	3 (8.11%)	
No	40 (93.00%)	34 (91.89%)	
Live birth rate of fresh cycle*			0.785
Yes	35 (81.40%)	30(81.08%)	
No	8 (18.60%)	7 (18.92%)	
Cumulative clinical pregnancy rate(per retrieval cycle)			0.097
Yes	114 (69.10%)	115 (77.70%)	
No	51 (30.90%)	33 (22.30%)	

Values are presented as mean ± standard deviation or number (percentage)

*Fisher’s exact test
